# Newly Diagnosed Anemia Increases Risk of Parkinson’s disease: A Population-Based Cohort Study

**DOI:** 10.1038/srep29651

**Published:** 2016-07-14

**Authors:** Chien Tai Hong, Yao Hsien Huang, Hung Yi Liu, Hung-Yi Chiou, Lung Chan, Li-Nien Chien

**Affiliations:** 1 Department of Neurology, Shuang Ho Hospital, Taipei Medical University, Taiwan; 2 Department of Neurology, School of Medicine, College of Medicine, Taipei Medical University, Taiwan; 3 School of Public Health, College of Public Health and Nutrition, Taipei Medical University, Taiwan; 4 School of Health Care Administration, College of Management, Taipei Medical University, Taiwan

## Abstract

Anemia and low hemoglobin have been identified to increase Parkinson’s disease (PD) risk. This population-based cohort study investigated PD risk in newly diagnosed anemic patients by using data from the Taiwan National Health Insurance Research Database. All newly diagnosed anemic patients (n = 86,334) without a history of stroke, neurodegenerative diseases, traumatic brain injury, major operations, or blood loss diseases were enrolled. A cohort of nonanemic controls, 1:1 matched with anemic patients on the basis of the demographics and pre-existing medical conditions, was also included. Competing risk analysis was used to evaluate PD risk in anemic patients compared with that in their matched controls. The adjusted hazard ratio (aHR) of PD risk in the anemic patients was 1.36 (95% confidence interval [CI]: 1.22–1.52, *p* < 0.001). Iron deficiency anemia (IDA) patients tended to exhibit a higher PD risk (aHR: 1.49; 95% CI: 1.24–1.79, *p* < 0.001). Furthermore, Iron supplement did not significantly affect the PD risk: the aHRs for PD risk were 1.32 (95% CI: 1.07–1.63, *p* < 0.01) and 1.86 (95% CI: 1.46–2.35, *p* < 0.001) in IDA patients with and without iron supplementation, respectively. The population-based cohort study indicated newly diagnosed anemia increases PD risk.

Parkinson’s disease (PD) is the second most common neurodegenerative disorder, with a prevalence of 0.3% in the general population and 1% in people older than 60 years^1^. No neuroprotective treatment for PD exists, but several approaches have been evaluated in clinical trials^2^,^3^. Delayed initiation of neuroprotection has been the main reason for the failure of these trials. Approximately 50% of the dopaminergic neurons in the substantia nigra are lost before the initial motor symptoms can be identified^4^,^5^. Moreover, according to the widely used Braak staging, Lewy body deposition initiated from the medulla and olfactory bulb occurs much earlier than the development of motor symptoms does^6^,^7^. Some prediagnostic symptoms of PD have been reported: rapid eye movement sleep behaviour disorder (RBD), olfactory dysfunction, and constipation occur before the motor symptoms of PD do^8^,^9^,^10^,^11^. However, with the current knowledge of these prediagnostic symptoms, we cannot predict the onset of PD in time to prevent disease progression.

Development of methods for timely prediction of PD is imperative, and additional prediagnostic PD symptoms and risk factors should be identified to strengthen the prediction and initiate appropriate neuroprotective treatment before most dopaminergic neurons in the substantia nigra are lost.

Erythrocytes are also affected in PD patients: the erythrocyte morphology of PD patients was more disturbed than that of healthy controls, and eryptosis (apoptosis of erythrocyte) was also enhanced^12^. A cohort from the United States demonstrated that anemia in the early stages of life is associated with PD development in the later stages of life. This association is most evident when anemia onset occurs 20–29 years before PD onset^13^, suggesting that anemia may precede the onset of PD motor symptoms. Therefore, the present population-based cohort study examined whether anemia and PD risk are associated.

## Results

### Patient selection and demographic data

**Figure 1:**
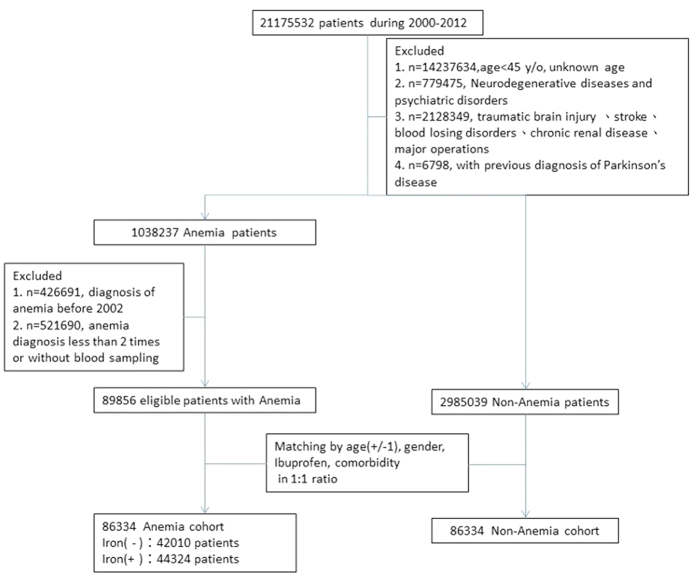
Patient selection flowchart. Asterisk (*) indicates anemia (ICD-9-CM: 280.1, 280.8, 280.9, 281.x, 284.01, 284.09, 284.8, 284.9, and 285.9).

The detailed process of patient selection is presented in Fig. 1. Table 1 shows the demographics and disease conditions of newly diagnosed anemic patients and nonanemic control patients. Of all the anemic patients (n = 86,334), 75.9% were women and the mean age was 56.4 ± 11.5 year-old. The prevalence of previous or current comorbidity among the anemic patients was 30.1%, 15.5%, 20.8%, and 5.1% for hypertension, diabetes, hyperlipidemia, and gout, respectively. Ibuprofen was prescribed for 25.1% of the anemic patients. After propensity score matching (PSM), the anemic patients and nonanemic controls exhibited nonsignificant differences in all the covariates.

### Anemia increases PD risk

**Figure 2:**
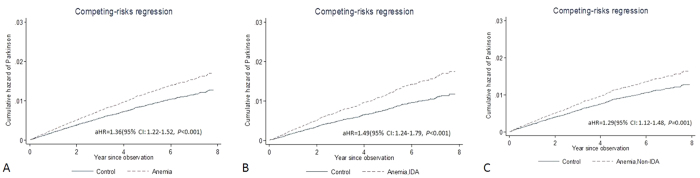
Cumulative hazards of PD based on competing risk regression analyses. (**A**) The aHR of developing PD was 1.36 (95% CI: 1.22–1.52, *p* < 0.001) for anemic patients. (**B**,**C**) The aHRs of developing PD for patients with and without iron IDA were 1.49 (95% CI: 1.24–1.79, *p* < 0.001) and 1.29 (95% CI: 1.12–1.48, *p* < 0.001), respectively.

Within a mean follow-up period of 6.6 years, newly diagnosed anemic patients were at a higher risk of PD than nonanemic controls were, after exclusion of patients who developed PD within 3 years of enrolment. The exclusion was made to prevent biases caused by simultaneous diagnosis of anemia and PD and by PD unmasked by anemia. The incidence rates of PD in the anemic patients and nonanemic controls were 2.38 (95% confidence interval [CI]: 2.20–2.57) and 1.69 (95% CI: 1.56–1.83) per 1,000 person-years, respectively, with an adjusted hazard ratio (aHR) of 1.36 (95% CI: 1.22–1.52, *p* < 0.001) for the anemic patients (Fig. 2A), based on the competing risk regression model. The aHRs for developing PD in the patients with and without iron deficiency anemia (IDA) were 1.49 (95% CI: 1.24–1.79, *p* < 0.001) and 1.29 (95% CI: 1.12–1.48, *p* < 0.001), respectively (Fig. 2B,C). The results obtained from competing risk models for stratified data showed similar significantly increased risk (Supplementary Table S1).

**Figure 3:**
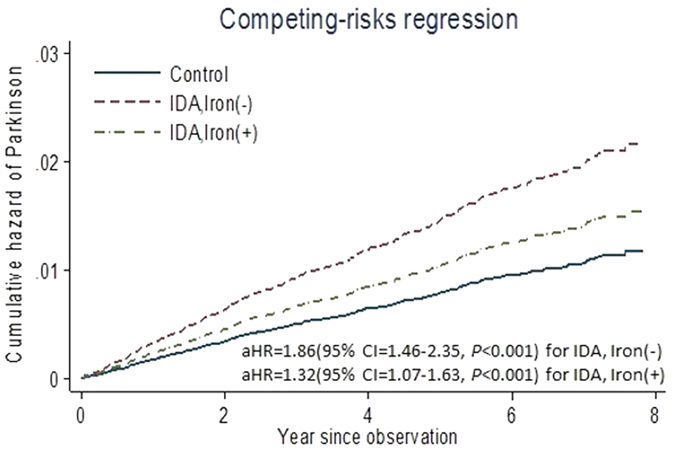
Cumulative hazards of PD for patients with IDA based on competing risk regression analyses. (**A**) The aHR of developing PD in IDA patients without substantial iron supplementation (more than 28 days) was 1.86 (95% CI: 1.46–2.35, *p* < 0.001). (**B**) The aHR of developing PD in IDA patients with more than 28 days of iron supplementation was 1.32 (95% CI: 1.07–1.63, *p* < 0.01).

Iron supplementation to IDA patients did not significantly affect PD risk (Fig. 3). The aHR of developing PD in IDA patients without iron supplementation was 1.86 (95% CI: 1.46–2.35, *p* < 0.001), and that in IDA patients with more than 28 days of iron supplementation was 1.32 (95% CI: 1.07–1.63, *p* < 0.01). Similar results were obtained from competing risk models for stratified data (Supplementary Table S2).

## Discussion

The present study demonstrated that newly diagnosed anemic patients might have a higher risk of developing PD 4 or more years after the initial anemia diagnosis. Of all the anemic patients, those with IDA tended to exhibit higher PD risk, which remained unaffected by iron supplementation.

The association between anemia and higher PD risk may not result from anemia alone. For instance, hereditary anemia types, such as sickle cell anemia and thalassemia, have not been identified as risk factors for PD, to the best of our knowledge. By contrast, several factors may account for the aforementioned association, such as oxidative stress, a major cause of PD^14^. Erythrocytes of PD patients demonstrate reduced superoxide dismutase and glutathione peroxidase activity and increased lipid peroxidation, both of which indicate increased oxidative stress^14^,^15^,^16^. Similar to the degeneration of dopaminergic neurons in the substantia nigra, erythrocytes exposed to excessive oxidative stress tend to undergo eryptosis, resulting in anemia^17^. The other possible pathogenetic connection between anemia and PD is α-synuclein, another key aetiology of PD. More than 99% of the α-synuclein in human blood originates from erythrocytes^18^, and the α-synuclein oligomer-to-total protein ratio of erythrocytes is higher in PD patients than in controls^19^.

Iron can be another crucial factor leading to the association between anemia and PD. Iron deficiency impairs erythropoiesis and enhances eryptosis, resulting in anemia, whereas increased iron deposition is noted in the substantia nigra of PD patients^20^,^21^,^22^. In addition, increased serum iron levels are associated with decreased PD risk, and high dietary iron intake reduces PD risk^23^,^24^. Iron maldistribution (i.e., improper distribution of iron within the body)^25^,^26^ may account for the iron deficit in the erythropoietic system and iron overload in the substantia nigra. Moreover, iron modulates dopamine synthesis and reuptake: tyrosine hydroxylase, an enzyme responsible for dopamine synthesis, is iron-dependent^27^, and iron deficiency impairs dopamine reuptake in a mouse model^28^.

The cellular vulnerability upon stress may account for the temporal interval between the onsets of anemia and PD. For instance, after exposure to excessive oxidative stress, neurons become more resistant because of their postmitotic characteristics^29^, and the lifespan of erythrocytes is only 120 days. Another possibility is that the onset of motor symptoms is not the most initial presentation of PD. The initiation of PD pathology occurs years before the degeneration of dopaminergic neurons in the substantia nigra^7^. Oxidative stress, accumulation of α-synuclein, and iron maldistribution may cause anemia as well as initiate PD pathology in the brain. The onset of anemia occurs earlier than that of the motor symptoms of PD does, similar to other nonmotor symptoms (such as RBD, anxiety, and constipation), which occur in the prediagnostic stage and precede PD diagnosis by 4–20 years^30^.

The strength of this study is that we identified newly diagnosed anemia as a potential biomarker of PD, after excluding anemia caused by chronic renal disease and several temporary blood loss-related anemia types (such as a pre-existing major operation or peptic ulcer); the exclusion enabled us to focus on anemia caused by impaired erythropoiesis and increased eryptosis. Anemia is common in the general population and can be easily detected through minimally invasive blood sampling. Anemia, along with other well-known risk factors for or prediagnostic symptoms of PD, such as RBD, olfactory dysfunction, and severe constipation^11^, can be used in the potential panel of biomarkers for predicting PD in the future. We also excluded patients with a ≤3-year interval between anemia and PD diagnoses to reduce the potential biases caused by simultaneous diagnosis of anemia and PD and by PD unmasked by anemia. Through the current approach, we confirmed the hypothesis that newly diagnosed anemia predicts PD risk. Finally, our PSM approach facilitated the selection of controls with baseline characteristics similar to those of our anemic patients for reducing the number of confounding factors.

This study had the following limitations. First, some anemic patients can be asymptomatic and thus not visit the clinic and obtain diagnosis; therefore, the incidence of PD in the nonanemic controls was probably overestimated because of the presence of these asymptomatic patients. Second, anemic patients are more likely to visit clinics; thus, they are more likely to be diagnosed with PD than nonanemic controls are. Third, the accuracy of the diagnosis of PD is of concern. Although Lee *et al.* had demonstrated that the accuracy of PD diagnosis in the NHIRD is 94.8% 31, however, the validation was conducted by medical review, which is not the standard way of diagnosing PD in most of studies. The misclassification of PD in National Health Insurance Research Database (NHIRD) by essential tremor or vascular Parkinson’sism may bias the true risk of anemia in developing PD. Considering the degree of misclassification of PD was the same in the anemia and nonanemic groups, it would result in underestimating the risk. Finally, a general limitation for all health insurance database research is the lack of information on some lifestyle risk factors and the raw data of examinations; data regarding factors, such as smoking, coffee consumption, well water consumption, and pesticide exposure, which can significantly affect incidence of PD^32^, were unavailable in the NHIRD and could not be adjusted for in the present study. In addition, unlike a previous study conducted by Savica *et al.*^13^, which analysed the hemoglobin levels and medical records, the present study relied on anemia diagnoses by clinicians, which may be less sensitive and delayed; nevertheless, this discrepancy in the data acquisition did not generate a conflicting result, further confirming the association between anemia and PD.

In summary, we demonstrated that newly diagnosed anemic patients might have a higher risk of developing PD 4 or more years after the initial anemia diagnosis, indicating the possible association between anemia and PD risk. Oxidative stress, α-synuclein accumulation, and iron deficiency or maldistribution may be the shared etiologies responsible for this association. Further research is warranted to identify the cause–consequence effect of these two disorders.

## Methods

### Ethics statement

This study was approved by the joint institutional review board of Taipei Medical University (approval N201509044). Confidentiality was ensured by abiding by data regulations of the Health and Welfare Data Science Center (HWDC), Ministry of Health and Welfare, Executive Yuan, Taiwan. The HWDC releases patient-level data to investigators only for research to protect patient privacy; moreover, the individual identifiers in these data are encrypted before release. Therefore, the requirement for informed consent from the study participants was exempted by the joint institutional review board. All the study methods were in accordance with the guidelines approved by the joint institutional review board and aforementioned governmental regulations.

### Data source

Patient-level data were derived from the NHIRD provided by the National Health Insurance Administration (NHIA) of Taiwan. The NHIRD is a nationwide claims-based database of the National Health Insurance (NHI) programme, a mandatory insurance programme that has provided most of the healthcare services in Taiwan and almost 30,000 prescription medications since 1995. In this study, data collected between 2000 and 2013 were used; we used data collected after the year 2000 because the rate of electronic claims was incomplete during the initial phases of NHI implementation. Therefore, the HWDC currently releases NHIRD data beginning from 2000 for both inpatient and outpatient visit claims. The NHIRD includes information on disease diagnoses (coded according to the International Classification of Diseases, Ninth Revision, Clinical Modification [ICD-9-CM]), treatment procedures, service dates, prescribed medications (classified according to the Anatomical Therapeutic Chemical [ATC] Classification System for Medications), reimbursement amounts, patient demographic information, and patient- and provider-encrypted identifiers. To verify the accuracy of the diagnoses and rationale for treatments, the NHIA routinely samples a portion of the NHI claims, and hospitals and clinics are penalised if they are found to provide unnecessary medical treatments.

### Study cohort

The study cohort consisted of newly diagnosed anemic patients aged 45 years or older, with at least two diagnosis claims for anemia (ICD-9-CM: 280.1, 280.8, 280.9, 281.x, 284.01, 284.09, 284.8, 284.9, and 285.9) or iron supplementary medication (ATC: B03A) between 2002 and 2009. Patients previously diagnosed with anemia in 2000 and 2001 were excluded. The purpose of setting this wash-out period is to make sure that all anemia patients were newly diagnosed. We then excluded patients with anemia secondary to blood loss, haemolytic disorders, pancytopenia, and vitamin deficiency-related anemia. In addition, we enrolled only those patients who received at least one blood test service claim within 3 months before the date of anemia diagnosis claim to ensure that the diagnosis of anemia was active. The first date of anemia diagnosis claim was considered the index date of anemia diagnosis. Patients were also excluded if they (1) had hereditary and degenerative diseases of the central nervous system (ICD-9-CM: 330–337) except for PD and psychiatric disorders (ICD-9-CM: 290–295); (2) had traumatic brain injury (ICD-9-CM: 800–804 and 850–854), which increases PD risk^33^; (3) had a history of PD before the index of anemia; (4) had cerebrovascular diseases (ICD-9-CM: 430–438), which may result in vascular parkinsonism; or (5) had some diseases or medical conditions that are likely to cause anemia, such as gastrointestinal bleeding disorders and chronic renal disease, and underwent major surgery (ICD-9 procedure codes: 01–05, surgery on the nervous system; 30, 32, and 33, surgery on the respiratory system; 35, 36.1–36.9, 37.5, 37.6, 38, and 39, surgery on the cardiovascular system; 40, surgery on the lymphatic system; and 42–54, surgery on the digestive system). An exclusion criterion (5) was used to exclude patients with anemia secondary to blood loss or general medical illness.

In the present study, the patients without an anemia diagnosis were referred to as nonanemic controls. For each anemic patient, we selected one nonanemic control through PSM, which aided in grouping each case and control with similar baseline characteristics but differences in anemia exposure^34^,^35^,^36^. PSM is commonly used in observational studies to reduce sample selection bias^37^,^38^,^39^. In this study, the propensity score was measured on the basis of age, sex, hypertension (ICD-9-CM: 401–405), diabetes (ICD-9-CM: 250), hyperlipidemia (ICD-9-CM: 272), gout (ICD-9-CM: 274), and renal disease (ICD-9-CM: 580–589). Ibuprofen (ATC: M01AE01) was also considered as a medication because it reduces PD risk^40^. Because the controls were nonanemic, they were assigned a date for pseudoanemia, corresponding to the index date of anemia of their matched anemic patients.

### Main outcome

The main outcome was PD diagnosis (ICD-9-CM: 332). To ensure the validity of diagnoses, patients with PD were selected only if they received PD medication (ATC: N04A and N04B) within the same visit for diagnosis because these medications confirmed that the disease presented as an active episode. To test the hypothesis that newly diagnosed anemia increases PD risk in the later stages of life, a 3-year window period was introduced between anemia and PD diagnoses, which enabled us to avoid including PD patients with simultaneous anemia and PD diagnoses as well as PD cases unmasked by anemia.

### Statistical analysis

The standardised difference was used to compare the mean of continuous and binary variables between anemic patients and matched controls. The standardised difference is a method used to measure the similarity of the baseline characteristics in propensity score-matched samples^34^,^36^,^38^ and represents the difference in the means of two groups in the units of standard deviation. Unlike significance testing, where the convention *p* < 0.05 denotes statistical significance, standardised differences of <0.10 likely denote a negligible imbalance between patients and their matched controls^36^,^38^.

To estimate the risk of anemia and PD in a population-based cohort study with a matched design, a stratified competing risk regression analysis should be applied. This is because the mortality rate of anemic patients is significantly higher that of nonanemic controls (17.75 vs. 9.62 per 1000 person-years). Without considering pre-PD deaths as a competing event, this study would more likely overestimate the association between anemia and PD. Therefore, we limited our analysis to consider only two competing events: PD development and pre-PD deaths; deaths occurring after PD development were not considered in this analysis. The follow-up period was calculated from the index date of anemia or pseudoanemia diagnosis after the completion of a 3-year washout period to (1) the date of PD diagnosis; (2) the date of death based on the Death Record Registry of Taiwan; or (3) December 31, 2013, for both the anemic patients and nonanemic controls not developing PD and pre-PD deaths. Rather than applying the competing risk regression for stratified data newly proposed by Zhou *et al.*^41^ and R package ‘crrSC’ developed in 2015, we used the ‘stcrreg’ in the Stata statistical software for estimating the association between anemia and PD. Stata’s stcrreg implements competing-risks regression according to Fine and Grey’s proportional subhazards model^42^ developed in 1999; it has become a regular package in STATA 11. The ‘stcrreg’ has been applied in numerous studies. By using this statistical package, we can obtain the estimations with no loss of validity, even without using matched analysis^43^. However, the results obtained using the competing risk model for the stratified data-based R package are presented in the Supplementary Data.

Subgroup analysis was conducted to select patients with IDA not secondary to blood loss (ICD-9-CM: 280.1, 280.8, and 280.9) for investigating whether iron connects the etiology of anemia and PD. In our specific group of patients, the effect of substantial iron supplementation (ATC: B03A; >28 days) was also evaluated. All analyses were performed using SAS/STAT 9.2 (SAS Institute Inc., Cary, NC, USA), STATA 12 (Stata Corp LP, College Station, TX, USA), and R (version 3.2.5 for Windows). A two-sided *p* value of <0.05 or a standardised difference of >0.1 was considered significant.

## Additional Information

**How to cite this article**: Hong, C. T. *et al.* Newly Diagnosed Anemia Increases Risk of Parkinson’s disease: A Population-Based Cohort Study. *Sci. Rep.* **6**, 29651; doi: 10.1038/srep29651 (2016).

## Supplementary Material

Supplementary Information

## Figures and Tables

**Table 1:** Baseline characteristics of anemic patients and propensity score-matched controls.

	Anemic patients n (%)	Nonanemic controls n (%)
Sample size	86,334(100)	86,334(100)
IDA	42,010(48.7)	
Non-IDA	44,324(51.3)	
Female	65,526(75.9)	65,526(75.9)
Age, mean (SD)	56.4(11.5) years	56.4(11.5) years
45–54	52,394(60.7)	51,483(59.6)
55–64	14,431(16.7)	14,781(17.1)
65–74	10,848(12.6)	10,971(12.7)
≥75	8,661(10.0)	9,099(10.5)
Ibuprofen use		
No	64,700(74.9)	64,700(74.9)
Yes	21,634(25.1)	21,634(25.1)
Comorbidity		
Hypertension	26,018(30.1)	26,037(30.2)
Diabetes	13,362(15.5)	13,247(15.3)
Hyperlipidemia	17,994(20.8)	18,001(20.9)
Gout	4,440(5.1)	4,395(5.1)

^$^Standardised difference represents the difference in the means of two groups in units of standard deviation, and a standardised difference of less than 0.10 likely denotes a negligible imbalance between anemic patients and their matched controls.

Abbreviation: IDA, iron deficiency anemia.

## References

[b1] de Lau L. M. L. & Breteler M. M. B. Epidemiology of Parkinson’s’s disease. The Lancet Neurology 5, 525, doi: 10.1016/S1474-4422(06)70471-9 (2006). 16713924

[b2] Schapira A. H., Olanow C. W., Greenamyre J. T. & Bezard E. Slowing of neurodegeneration in Parkinson’s’s disease and Huntington’s disease: future therapeutic perspectives. Lancet 384, 545, doi: 10.1016/s0140-6736(14)61010-2 (2014). 24954676

[b3] Kalia L. V., Kalia S. K. & Lang A. E. Disease-modifying strategies for Parkinson’s’s disease. Mov Disord 30, 1442, doi: 10.1002/mds.26354 (2015). 26208210

[b4] Olanow C. W., Kieburtz K. & Schapira A. H. Why have we failed to achieve neuroprotection in Parkinson’s’s disease? Ann Neurol 64 Suppl 2, S101, doi: 10.1002/ana.21461 (2008). 19127580

[b5] Schapira A. H. Progress in neuroprotection in Parkinson’s’s disease. Eur J Neurol 15 Suppl 1, 5, doi: 10.1111/j.1468-1331.2008.02055.x (2008). 18353131

[b6] Braak H., Sastre M., Bohl J. R., de Vos R. A. & Del Tredici K. Parkinson’s’s disease: lesions in dorsal horn layer I, involvement of parasympathetic and sympathetic pre- and postganglionic neurons. Acta Neuropathol 113, 421, doi: 10.1007/s00401-007-0193-x (2007). 17294202

[b7] Braak H. *et al.* Staging of brain pathology related to sporadic Parkinson’s’s disease. Neurobiol Aging 24, 197 (2003). 12498954 10.1016/s0197-4580(02)00065-9

[b8] Goldman J. G. & Postuma R. Premotor and nonmotor features of Parkinson’s’s disease. Curr Opin Neurol 27, 434, doi: 10.1097/wco.0000000000000112 (2014). 24978368 PMC4181670

[b9] Pont-Sunyer C. *et al.* The onset of nonmotor symptoms in Parkinson’s’s disease (the ONSET PD study). Mov Disord 30, 229, doi: 10.1002/mds.26077 (2015). 25449044

[b10] Adler C. H. Premotor symptoms and early diagnosis of Parkinson’s’s disease. Int J Neurosci 121 Suppl 2, 3, doi: 10.3109/00207454.2011.620192 (2011). 22035024

[b11] Noyce A. J., Lees A. J. & Schrag A.-E. The prediagnostic phase of Parkinson’s’s disease. Journal of Neurology, Neurosurgery & Psychiatry , doi: 10.1136/jnnp-2015-311890 (2016). PMC4975823 26848171

[b12] Pretorius E. *et al.* Eryptosis as a marker of Parkinson’s’s disease. *Aging* (*Albany NY*) 6, 788 (2014). 25411230 10.18632/aging.100695 PMC4247384

[b13] Savica R. *et al.* Anemia or low hemoglobin levels preceding Parkinson’s disease: A case-control study. Neurology 73, 1381, doi: 10.1212/WNL.0b013e3181bd80c1 (2009). 19858460 PMC2769554

[b14] Johannsen P., Velander G., Mai J., Thorling E. B. & Dupont E. Glutathione peroxidase in early and advanced Parkinson’s’s disease. J Neurol Neurosurg Psychiatry 54, 679 (1991). 1940936 10.1136/jnnp.54.8.679 PMC1014468

[b15] Urakami K. *et al.* Decreased superoxide dismutase activity in erythrocyte in Parkinson’s’s disease. Jpn J Psychiatry Neurol 46, 933, (1992). 1304619 10.1111/j.1440-1819.1992.tb02863.x

[b16] Kilinc A., Yalcin A. S., Yalcin D., Taga Y. & Emerk K. Increased erythrocyte susceptibility to lipid peroxidation in human Parkinson’s’s disease. Neurosci Lett. 87, 307 (1988). 3380350 10.1016/0304-3940(88)90467-3

[b17] Lang F., Abed M., Lang E. & Foller M. Oxidative stress and suicidal erythrocyte death. Antioxid Redox Signal 21, 138, doi: 10.1089/ars.2013.5747 (2014). 24359125

[b18] Barbour R. *et al.* Red blood cells are the major source of alpha-synuclein in blood. Neurodegener Dis. 5, 55, doi: 10.1159/000112832 (2008). 18182779

[b19] Wang X., Yu S., Li F. & Feng T. Detection of alpha-synuclein oligomers in red blood cells as a potential biomarker of Parkinson’s’s disease. Neurosci Lett. 599, 115, doi: 10.1016/j.neulet.2015.05.030 (2015). 25998655

[b20] Martin W. R., Wieler M. & Gee M. Midbrain iron content in early Parkinson’s disease: a potential biomarker of disease status. Neurology 70, 1411, doi: 10.1212/01.wnl.0000286384.31050.b5 (2008). 18172063

[b21] Dexter D. T. *et al.* Increased nigral iron content and alterations in other metal ions occurring in brain in Parkinson’s’s disease. J Neurochem 52, 1830 (1989). 2723638 10.1111/j.1471-4159.1989.tb07264.x

[b22] Sofic E. *et al.* Increased iron (III) and total iron content in post mortem substantia nigra of Parkinson’sian brain. J Neural Transm 74, 199 (1988). 3210014 10.1007/BF01244786

[b23] Pichler I. *et al.* Serum iron levels and the risk of Parkinson’s disease: a Mendelian randomization study. PLoS Med. 10, e1001462, doi: 10.1371/journal.pmed.1001462 (2013). 23750121 PMC3672214

[b24] Miyake Y. *et al.* Dietary intake of metals and risk of Parkinson’s’s disease: a case-control study in Japan. J Neurol Sci. 306, 98, doi: 10.1016/j.jns.2011.03.035 (2011). 21497832

[b25] Cabantchik Z. I., Munnich A., Youdim M. B. & Devos D. Regional siderosis: a new challenge for iron chelation therapy. Frontiers in Pharmacology 4, 167, doi: 10.3389/fphar.2013.00167 (2013). 24427136 PMC3875873

[b26] Kaneko J. J. In Clinical Biochemistry of Domestic Animals (Third Edition) 649 (Academic Press, 1980).

[b27] Roberts K. M. & Fitzpatrick P. F. Mechanisms of tryptophan and tyrosine hydroxylase. IUBMB Life 65, 350, doi: 10.1002/iub.1144 (2013). 23441081 PMC4270200

[b28] Nelson C., Erikson K., Piñero D. J. & Beard J. L. *In Vivo* Dopamine Metabolism Is Altered in Iron-Deficient Anemic Rats. The Journal of Nutrition 127, 2282 (1997). 9405575 10.1093/jn/127.12.2282

[b29] Herrup K. & Yang Y. Cell cycle regulation in the postmitotic neuron: oxymoron or new biology? Nat Rev Neurosci 8, 368 (2007). 17453017 10.1038/nrn2124

[b30] Savica R., Rocca W. A. & Ahlskog J. WHen does Parkinson’s disease start? Archives of Neurology 67, 798, doi: 10.1001/archneurol.2010.135 (2010). 20625084

[b31] Lee Y. C. *et al.* Discontinuation of statin therapy associates with Parkinson’s disease: a population-based study. Neurology 81, 410, doi: 10.1212/WNL.0b013e31829d873c (2013). 23884037

[b32] Kieburtz K. & Wunderle K. B. Parkinson’s’s disease: evidence for environmental risk factors. Mov Disord 28, 8, doi: 10.1002/mds.25150 (2013). 23097348

[b33] Gardner R. C. *et al.* Traumatic brain injury in later life increases risk for Parkinson’s disease. Ann Neurol 77, 987, doi: 10.1002/ana.24396 (2015). 25726936 PMC4447556

[b34] Rosenbaum P. R. & Rubin D. B. Constructing a Control Group Using Multivariate Matched Sampling Methods That Incorporate the Propensity Score. The American Statistician 39, 33, doi: 10.2307/2683903 (1985).

[b35] Rosenbaum P. R. & Rubin D. B. The central role of the propensity score in observational studies for causal effects. Biometrika 70, 41, doi: 10.1093/biomet/70.1.41 (1983).

[b36] Faries D. E. SAS Institute & Books24x7 Inc. (SAS Institute, Cary, N.C., 2010).

[b37] Austin P. C. A critical appraisal of propensity-score matching in the medical literature between 1996 and 2003. Stat Med. 27, 2037, doi: 10.1002/sim.3150 (2008). 18038446

[b38] Austin P. C., Grootendorst P. & Anderson G. M. A comparison of the ability of different propensity score models to balance measured variables between treated and untreated subjects: a Monte Carlo study. Stat Med. 26, 734, doi: 10.1002/sim.2580 (2007). 16708349

[b39] Guo S. & Fraser M. W. Propensity score analysis: statistical methods and applications . (Sage Publications, 2010).

[b40] Rees K. *et al.* Non-steroidal anti-inflammatory drugs as disease-modifying agents for Parkinson’s’s disease: evidence from observational studies. *Cochrane Database Syst Rev*. Cd008454, doi: 10.1002/14651858.CD008454.pub2 (2011). 22071848 PMC13579550

[b41] Zhou B., Latouche A., Rocha V. & Fine J. Competing risks regression for stratified data. Biometrics 67, 661, doi: 10.1111/j.1541-0420.2010.01493.x (2011). 21155744 PMC3431205

[b42] Fine J. P. & Gray R. J. A Proportional Hazards Model for the Subdistribution of a Competing Risk. Journal of the American Statistical Association 94, 496, doi: 10.2307/2670170 (1999).

[b43] Pearce N. Analysis of matched case-control studies. BMJ. 352 (2016). 10.1136/bmj.i969 PMC4770817 26916049

