# Supplementary material for: Newly Diagnosed Anemia Increases Risk of Parkinson’s disease: A Population-Based Cohort Study
Source: Sci Rep. 2016 Jul 14;6:29651. doi: 10.1038/srep29651 (PMC4944159; doi:10.1038/srep29651)
Supplement: Supplementary Information [file srep29651-s1.doc]

Title Page:

Newly Diagnosed Anemia Increases Risk of Parkinson’s Disease: A Population-Based Cohort Study

Chien Tai Hong, MD, PhD^1,2^; Yao Hsien Huang, MD^1,2^; Hung Yi Liu, BS^3^; Hung-Yi Chiou, PhD^3^; Lung Chan, MD, PhD^1,2*^; Li-Nien Chien, PhD^4*^

*These authors contributed equally to the manuscript

1. Department of Neurology, Shuang Ho Hospital, Taipei Medical University, Taiwan
2. Department of Neurology, School of Medicine, College of Medicine, Taipei Medical University, Taiwan
3. School of Public Health, College of Public Health and Nutrition, Taipei Medical University, Taiwan
4. School of Health Care Administration, College of Management, Taipei Medical University, Taiwan

Corresponding authors:

Lung Chan, Department of Neurology, Shuang Ho Hospital, Taipei Medical University, Taiwan. Tel: +886-2-22490088ext8112

Address: No. 291, Zhongzheng Rd, Zhonghe District, New Taipei City 23561, Taiwan

E-mail: cjustinmd@gmail.com

Li-Nien Chien, School of Health Care Administration, College of Management, Taipei Medical University, Taiwan. Tel: +886-2-2736-1661ext3628

Address: 250 Wuxing Street, Taipei City 110, Taiwan

E-mail: lnchien@tmu.edu.tw

Supplementary data

| Supplementary Table 1: Association between anemia and Parkinson’s disease risk based on competing risk models for stratified data | | | |
| --- | --- | --- | --- |
|  | Hazard ratio | 95% confidence interval | *p* value |
| Overall (anaemic vs. nonanemic) | 1.46 | 1.31–1.63 | <0.001 |
| IDA vs nonanemic | 1.60 | 1.35–1.90 | <0.001 |
| Non-IDA vs nonanemic | 1.37 | 1.19–1.58 | <0.001 |

Abbreviation: IDA, iron deficiency anemia. Reference groups were nonanemic controls

| Supplementary Table 2: Association between iron supplementation in IDA patients and Parkinson’s disease risks based on competing risk models for stratified data | | | |
| --- | --- | --- | --- |
|  | Hazard ratio | 95% confidence interval | *p* value |
| Nonanemic controls | 1.00 | Ref. |  |
| IDA, iron (+) | 1.87 | 1.40–2.50 | <0.001 |
| IDA, iron (−) | 1.45 | 1.17–1.80 | <0.001 |

Abbreviation: IDA, iron deficiency anemia.
